# Comparison of Artificial Intelligence Models for Automatic Segmentation of the Mandibular Canals and Branches

**DOI:** 10.1016/j.identj.2026.109427

**Published:** 2026-02-06

**Authors:** Hanyang Man, Shikun Ma, Huifeng Luo, Bing Wang, Jinlong Shao, Shaohua Ge, Hom-Lay Wang

**Affiliations:** a Department of Periodontology, School and Hospital of Stomatology, Cheeloo College of Medicine, Shandong University and Shandong Key Laboratory of Oral Tissue Regeneration and Shandong Engineering Research Center of Dental Materials and Oral Tissue Regeneration and Shandong Provincial Clinical Research Center for Oral Diseases, Jinan, Shandong, China; b School of Computer Science and Technology, Shandong University, Qingdao, Shandong, China; c Department of Periodontics and Oral Medicine, University of Michigan School of Dentistry, Ann Arbor, Michigan, USA

**Keywords:** Artificial intelligence, Mandibular canal, Mental foramen, Mandibular incisive canal

## Abstract

**Objectives:**

This study aimed to compare and improve the performance of three deep learning models, i.e., U-Net Transformer (UNETR), Swin UNETR, and 3D UX-Net, for the segmentation of the mandibular canal and its branches.

**Materials and methods:**

A dataset of 173 cone beam computed tomography (CBCT) scans was used for training, validation, and testing. The mandibular canals and branches were segmented manually and by the three AI models. A postprocessing module based on anatomical characteristics was then applied to improve model performance. Evaluations were conducted using Dice similarity coefficient (DSC), intersection over union (IoU), 95th Percentile Hausdorff Distance (HD95), average symmetric surface distance (ASSD), precision, and recall.

**Results:**

All models efficiently segmented the mandibular, incisive, and mental canals, operating at least 25 times faster than manual annotation. Both 3D UX-Net and Swin UNETR consistently outperformed the UNETR network across most metrics, with 3D UX-Net demonstrating a slight performance advantage over Swin UNETR in terms of DSC, IoU, and recall. Furthermore, the anatomically-based postprocessing module significantly improved the metrics for all models. Ultimately, the 3D UX-Net with postprocessing achieved the highest accuracy, with mean values of 0.788 (DSC), 0.652 (IoU), 0.23 mm (HD95), 0.083 mm (ASSD), 72.7% (precision), and 87.0% (recall).

**Conclusion:**

3D UX-Net and Swin UNETR are superior to the UNETR for segmenting small dental structures. Between the two, 3D UX-Net demonstrated statistically significant improvements in overlap and recall. Furthermore, the performances of these models can be significantly enhanced by applying postprocessing strategies based on anatomical characteristics.

## Introduction

Artificial intelligence (AI) is playing a transformative role in modern dentistry, revolutionising fields ranging from diagnostics and treatment planning to patient care.^1^ While AI offers immense promise, its most critical application lies in enhancing precision during surgical procedures.^2^ In this context, cone beam computed tomography (CBCT) has become a common tool for evaluating anatomic structures to prevent injuries during dental implantation, tooth extraction, and tumour resection.^3^ Specifically, the precise delineation of the mandibular canals and their fine terminal branches, such as the mandibular incisive and mental canals, is essential to protect the neurovascular bundle.^4^ However, current clinical workflows rely heavily on manual segmentation or semi-automated reference points.^5^ These methods are labour-intensive, time-consuming, and heavily dependent on clinician experience, creating a bottleneck that fails to meet the growing demand for efficient oral diagnostics.^6^ To address these limitations, advancements in deep learning, particularly in automated image segmentation, have emerged as a promising solution.^7^^,^^8^

Convolutional neural networks (CNNs) have shown considerable promise in image recognition and generation, and have shown feasibility for the automatic identification of the mandibular canal. Early CNN architectures, such as U-Net, have been successfully applied to image segmentation tasks.^9^ Subsequent improvements to U-Net have enhanced the recovery of image details in segmentation, largely through the use of an encoder-decoder structure with skip connections that link corresponding layers of the encoder and decoder.^10^ In this architecture, the encoder progressively reduces spatial resolution to learn contextual representations, while the decoder incrementally up-samples these representations to produce pixel/voxel-level predictions.^10^ Despite these advancements, standard CNN-based U-Net models often struggle to capture global context and long-range spatial dependencies, which can substantially limit segmentation performance, particularly in more challenging tasks.

Besides CNNs, the success of transformer-based models in natural language processing (NLP) has prompted their adoption in image processing.[11, 12, 13 U-Net Transformer (UNETR) reformulated the task of volumetric (3D) medical image segmentation as a sequence-to-sequence prediction problem and used a U-shaped architecture to integrate a pure transformer as the encoder to learn the sequential representation of input volumetric data.^14^ Moreover, the Swin UNETR architecture employs the Swin Transformer as the encoder.^15^^,^^16^ The Swin Transformer, or Shifted Window Transformer, incorporates hierarchical representation learning and attention mechanisms via shifted windows. This approach helps to reduce computational complexity while capturing local and global features more efficiently.

In contrast to these transformer-encoder networks, 3D UX-Net takes a different approach. 3D UX-Net is presented as a lightweight 3D CNN architecture that uses ConvNet modules.^17^ In its encoder section, 3D UX-Net applies a large convolutional kernel to map the raw input image to a low-dimensional latent space with reduced resolution, followed by the main feature extraction stages. Multiscale outputs from the encoder are linked to a ConvNet-based decoder through long skip connections, forming a U-shaped network similar to the U-Net.

UNETR, Swin UNETR, and 3D UX-Net have become popular baseline models for further exploration in medical image segmentation. However, these models have primarily been tested on larger anatomical structures, such as the brain and abdominal organs; very little research has been conducted on intricate structures like the mandibular canals with their branches. Nevertheless, these models still face challenges in clinical applications, primarily because most datasets only label the portion of the mandibular canal anterior to the mental foramen. Therefore, further training and fine-tuning of models are necessary. The continuous anatomical features of the canals offer a possibility to further refine the models.

To this end, this study aimed to compare the efficacy of automatic segmentation of mandibular canals and their branches by three potential AI models, ie, UNETR, Swin UNETR, and 3D UX-Net, and to explore whether postprocessing considering the anatomic characteristics of the canals could improve the model performances.

## Materials and methods

### Study design

Data from 343 full-head CBCT images in the Stomatological Hospital of Shandong University from October 2022 to July 2023 were collected. Following the recommendations in a recent comprehensive review of dental AI guidelines by Khurshid et al,^18^ we adopted the Checklist for Artificial Intelligence in Medical Imaging (CLAIM) guidelines as our primary reporting framework.

### Inclusion and exclusion criteria

The inclusion criteria were as follows: (1) patients aged 18 years or older; (2) patients whose bilateral mandibular canals were clearly visible.

The exclusion criteria were as follows: (1) images affected by implants, orthodontic appliances, filling materials, missing teeth, large cysts, benign or malignant tumours, mandibular fractures, or severe osteomyelitis affecting the canal region; (2) images blurred due to technical issues during imaging or motion artefacts.

### Dataset characteristics

All maxillofacial volumes were acquired using a NewTom 5G device (QR s.r.l., Verona, Italy) with standardised parameters: 110 kVp, approximately 6 mAs, and an isotropic voxel size of 0.30 mm. To ensure the model’s robustness across varying clinical protocols, the dataset incorporated two field-of-view (FOV) settings, including 164 large-view (16 × 18 cm) and 9 small-view (12 × 8 cm) scans.

Following the application of inclusion and exclusion criteria, 173 scans were selected. The sample size (*N* = 173) was benchmarked against the MICCAI 2023 ToothFairy Challenge, a recognised gold standard in dental image segmentation that utilised approximately 150 densely annotated volumes. While our total sample size is numerically comparable to this benchmark, the complexity of our dataset is inherently higher, as it includes both the primary mandibular canal and its intricate terminal branches. This increased anatomical detail necessitates higher annotation precision, making the current sample size sufficient for training a robust, high-granularity model. To ensure clinical representativeness, all subsets were verified for anatomical variability and image quality by dental specialists (B.W. and J.S.).

### Data annotation and preprocessing

To establish the accuracy of mandibular canal segmentation, a fully manual annotation method was employed.^19^ The entire dataset was segmented by two trained researchers (H.M. and H.L.) using 3D Slicer (Version 5.5.0; www.slicer.org). To verify the reliability of these manual annotations, an inter-rater reliability analysis was conducted on a randomly selected subset of 10 CBCT volumes segmented independently by both researchers. Agreement was quantified using Cohen's Kappa coefficient. In instances of discrepancy regarding canal boundaries or continuity, a consensus was reached through adjudication by a senior periodontist and radiologist (J.S.). The manual segmentation protocol demonstrated high reproducibility. Quantitative analysis yielded a mean inter-rater Cohen's Kappa of 0.903 ± 0.006. Intrarater consistency was assessed by having each researcher re-segment the same subset after a 2-week interval, yielding Kappa scores of 0.951 ± 0.022 (H.M.) and 0.935 ± 0.020 (H.L.). According to the guidelines established by Landis and Koch,^20^ these values indicate ‘almost perfect’ agreement, confirming the robustness of the ground truth labels used in this study.

Following annotation, image intensity was preprocessed to isolate the region of interest. Based on a statistical analysis of the ground truth regions, intensity values were clipped to the 1st and 99th percentiles of the Hounsfield Units (HU) observed within the marked areas. This thresholding strategy preserves essential details of the skull and mandibular canal while enhancing contrast against surrounding bone tissue. Finally, conventional data augmentation techniques were applied during training to enhance the model’s generalisation capability and robustness against variations in scan orientation and quality. After intensity normalisation, the following random transformations were applied: Rotation: ± 12°, Scaling: ± 0.1x, HU value shift: ± 0.1.

### AI model processing

A total of 128 CBCTs were assigned to the training set, 20 CBCTs to the validation set, and 25 CBCTs to the test set. The tasks were completed by either a server or personal workstations, as shown in Table 1.

**Table 1:** The training and inference environments of the AI modelling.

Component	Training environments	Inference environments
CPU	Intel(R) Xeon(R) Silver 4214R CPU x12 vCPU	AMD Ryzen 5 5600G CPU
GPU	Nvidia RTX 4090(24GB) * 1	Nvidia RTX 2080 Ti(22GB) * 1
RAM	90GB DDR4 RAM	16GB DDR4 RAM
Linux distribution	Ubuntu 22.04.1 LTS	Ubuntu 22.04.04 LTS
Linux kernel version	Linux version 5.4.0	Linux Version 6.5.0
Python version	Python 3.10.14	Python 3.11.5
Torch version	Torch 2.3.1	Torch 2.4.0

**Fig. 1:**
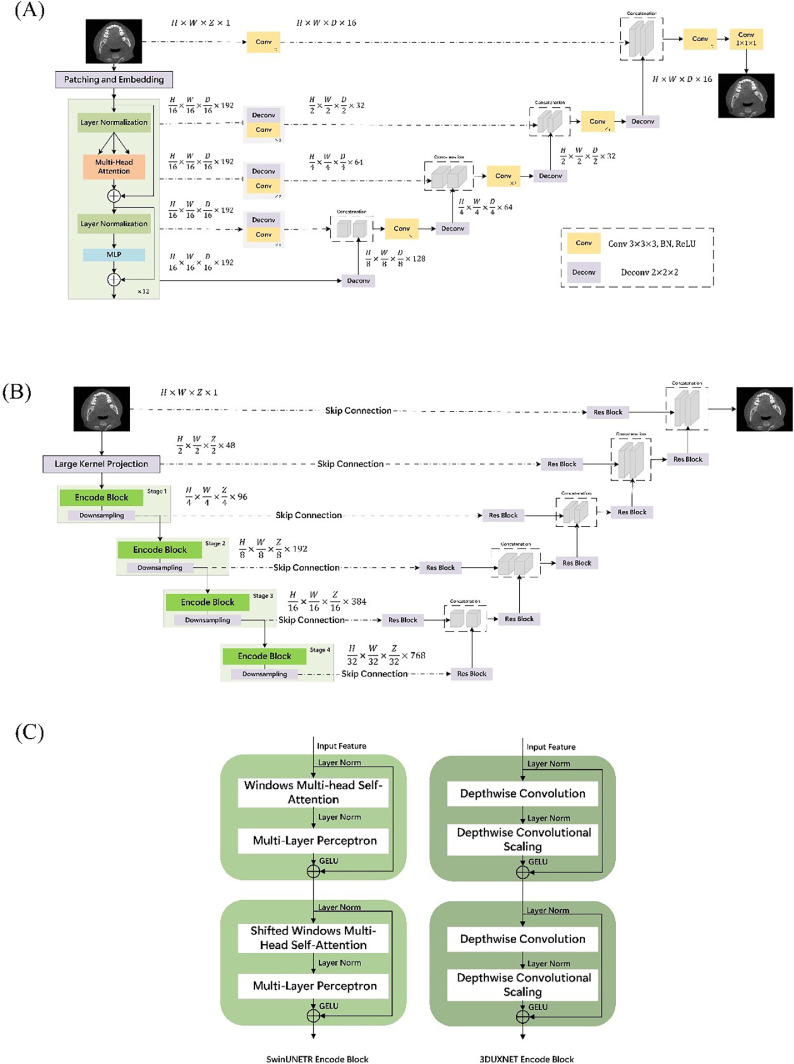
The network architectures of the AI models. (A) UNETR, (B) Swin UNETR and 3D UX-Net, (C) comparison of the encoder modules for Swin UNETR and 3D UX-Net.

The data were processed using three distinct deep learning models: UNETR, Swin UNETR, and 3D UX-Net. All three employ a U-shaped encoder-decoder architecture for volumetric medical image segmentation, but they differ in their core feature extraction methods. UNETR employs a pure vision transformer as its encoder, dividing the volumetric image into 3D patches processed by 12 stacked self-attention layers to capture global feature dependencies. Unlike traditional CNNs, UNETR’s encoder maintains a constant feature resolution throughout its depth, linking intermediate transformer outputs to a convolutional decoder via skip connections (Figure 1A). The global-sense ability brings a significantly high computation cost. Swin UNETR introduces a hierarchical Swin Transformer encoder with shifted window-based self-attention mechanisms, which addresses the high computational complexity of global attention. It utilises shifted window-based self-attention mechanisms, enabling the model to compute local attention within nonoverlapping windows while facilitating cross-window connections. This hierarchical design allows for efficient multiscale feature representation, and its decoder aggregates these features through skip connections to reconstruct the full-resolution mask (Figure 1B). In contrast, 3D UX-Net is a pure 3D convolutional neural network inspired by ConvNeXt. Instead of self-attention, it uses large-kernel depthwise convolutions (eg, 7 × 7 × 7) to provide a receptive field comparable to Transformers. This approach allows the model to extract long-range structural information while maintaining the inductive biases and computational efficiency of CNNs. At each stage of the encoder, multiscale outputs are linked to a ConvNet-based decoder via long skip connections, forming a U-shaped network similar to a U-Net that facilitates segmentation (Figure 1B). Interestingly, the 3D UX-Net's encoder directly adapts the Swin Transformer’s hierarchical layout but replaces the Multi-Head Self-Attention module with Depthwise Convolution and Depthwise Convolutional Scaling modules (Figure 1C), highlighting its distinct, convolution-based methodology while achieving performance comparable with ViT.

The hyperparameters for training each model are shown in Table 2. Due to hardware constraints, the batch size was set to 2, as higher values would lead to excessive memory consumption. To identify the optimal learning rate for rapid convergence, we conducted a series of preliminary experiments. Specifically, we employed a search strategy where the learning rate was successively doubled and evaluated over 5000 training steps while monitoring the loss curves. Based on the observed trends, we narrowed down the range and tested equidistant intervals within this scope to determine the final learning rate. To supervise the training process, the Dice Loss (*L_Dice_*) was employed as the objective function, which is specifically designed to handle the class imbalance between the narrow mandibular canal and the vast background. The loss function is defined as $L_{Dice}=1-DSC$, where DSC represents the Dice Similarity Coefficient. The detailed mathematical formulation of DSC is provided in the next section.

**Table 2:** The hyperparameters for training AI models.

	Batch size	Crop size	Learning rate	Optimiser	MaxIteration
UNETR	2	2	0.004	AdamW	30 000
Swin UNETR	2	2	0.005	AdamW	30 000
3D UX-Net	2	2	0.0025	AdamW	30 000

### Postprocessing

To mitigate isolated noise and artefacts inherent in the initial segmentation, a multistage postprocessing protocol was applied, grounded in the anatomical characteristics of the mandibular canals.^21^^,^^22^ First, a morphological closing operation (dilation followed by erosion) was performed to bridge small discontinuities and ensure the structural integrity of the segmented canals. A spherical structuring element with a radius of 1 voxel $\left(B_{r}=1\right)$ was utilised for these operations. Subsequently, connected component analysis was employed to eliminate spurious artefacts. To differentiate the mandibular canals from noise, a volume-based filtering strategy was prioritised over simple top-k selection. A volume threshold of 900 voxels (24.3 mm^3^) was established; components falling below this threshold were discarded, effectively isolating the anatomically significant structures corresponding to the bilateral canals. Finally, to correct for potential boundary enlargement induced by the morphological dilation and to ensure precise anatomical fidelity, a density-based refinement was applied. The segmentation boundaries were realigned with the inherent density of the canal by referencing the HU intensity percentiles $\left(P_{HU}=25\%\right)$ from the original image. This step ensured accurate delineation of the canal margins relative to the surrounding bone.

### Model evaluation

The average processing time by either manual segmentation or AI models was recorded to evaluate the model's efficiency. The segmentation accuracy was quantitatively evaluated using six distinct metrics:

• Dice similarity coefficient (DSC): This metric measures the volumetric overlap between the predicted segmentation and the ground truth, with a value ranging from 0 to 1, where 1 indicates a perfect overlap.^23^^,^^24^ The formula used is shown in Equation 1.
  (1) $$DSC=\frac{2TP}{2TP+FP+FN}$$
• Intersection over union (IoU): Also known as the Jaccard Index, IoU quantifies the region-based overlap between the predicted and true segmentation masks, with values ranging from 0 to 1.^25^ The calculation is outlined in Equation 2.
  (2) $$IoU=\frac{TP}{TP+FP+FN}$$
• 95th Percentile Hausdorff Distance (HD95): HD95 measures the 95th percentile of the maximum surface distances between the ground truth surface, S(T), and the predicted surface, S(P).^26^ This metric is reported in millimetres and is detailed in Equation 3.
  (3) HD95=max{maxp95d(t,S(P),maxp95d(p,S(T)|t∈S(T),p∈S(P)))}
• Average symmetric surface distance (ASSD): The ASSD computes the mean surface distance between the ground truth and predicted surfaces.^27^ This metric, also in millimetres, provides a symmetrical measure of surface similarity and is described in Equation 4.
  (4) $$ASSD=\frac{\sum_{t\in S\left(T\right)}d\left(t,S\left(P\right)\right)+\sum_{p\in S\left(P\right)}d\left(t,S\left(T\right)\right)}{\left|S\left(T\right)\right|+\left|S\left(P\right)\right|}$$
• Precision: This metric reflects the specificity of the model's predictions, indicating the proportion of correct positive predictions.^28^ The formula for Precision is given in Equation 5.
  (5) $$Precision=\frac{TP}{TP+FP}$$
• Recall: Also known as sensitivity, Recall measures the completeness of the model's detection by indicating the proportion of true positives that were correctly identified.^28^ The formula is provided in Equation 6.
  (6) $$Recall=\frac{TP}{TP+FN}$$

In the aforementioned equations, *TP* represents a true positive, *TN* a true negative, *FP* a false positive, and *FN* a false negative. For the spatial metrics, *t* and *p* denote the coordinates of a voxel in the ground truth set (*T*) and predicted set (*P*), respectively. The operations S(T) and S(P) extract the surface voxels from the sets of ground truth and predicted voxels, respectively.

### Statistical analysis

All data were expressed as mean ± standard deviation (SD) and statistically analysed using GraphPad Prism (9.0) software. All continuous variables were expressed as mean ± standard deviation (SD). The normality of the data distribution was evaluated using the Shapiro-Wilk test. For demographic characteristics, comparisons among the training, validation, and test sets were performed using one-way analysis of variance (ANOVA) for continuous variables (age) and the Chi-square test for categorical variables (sex). For model performance evaluation, a two-way repeated measures ANOVA was utilised to assess the main effects of model architecture (UNETR, Swin UNETR, 3D UX-Net) and postprocessing (origin vs postprocessed), as well as their interactions. Upon finding significant interaction effects, post-hoc pairwise comparisons were conducted. Specifically, Tukey’s multiple comparison test was used to analyse differences between models within the same processing stage, while Šídák’s multiple comparison test was employed to evaluate the effect of postprocessing within each model. A *P*-value < .05 was considered statistically significant.

## Results

### Demographic characteristics

A total of 173 CBCT scans were included, representing 82 males and 91 females with a mean age of 40.3 ± 22.3 years. The dataset was randomly partitioned into training (*n* = 128; 63 males, 65 females), validation (*n* = 20; 8 males, 12 females), and test (*n* = 25; 11 males, 14 females) sets, maintaining an approximate 7:1:1 ratio. Statistical analysis confirmed the demographic homogeneity of the partitions. There were no significant differences in sex distribution (*P* > .05) or mean age across the three cohorts (training: 40.2 ± 22.2 years; validation: 36.3 ± 22.1 years; test: 44.2 ± 23.2 years; *P* > .05). This balanced allocation ensures that the performance metrics derived from the test set serve as an unbiased estimate of the model’s generalisation capabilities.

### Segmentation time

Manual segmentation was the most time-consuming approach, requiring nearly an hour per case (Table 3). In contrast, all AI models offered substantial acceleration, with even the slowest method operating within minutes, which represents at least a 25-fold efficiency gain. Among the AI models, UNETR was the fastest, with an average processing time of 38 seconds, followed by Swin UNETR at 96 s and 3D UX-Net at 121 seconds. The postprocessing procedure added approximately 20 seconds to the processing time for all models.

**Table 3:** The segmentation time of different methods.

	Time (s)		Ratio to manual
	Mean	SD	
Manual	3562	649	1
UNETR	38	12	1/94
UNETR-Post	57	13	1/63
Swin UNETR	96	31	1/37
Swin UNETR-Post	114	31	1/31
3D UX-Net	121	39	1/29
3D UX-Net-Post	142	39	1/25

### Model performances visually

**Fig. 2:**
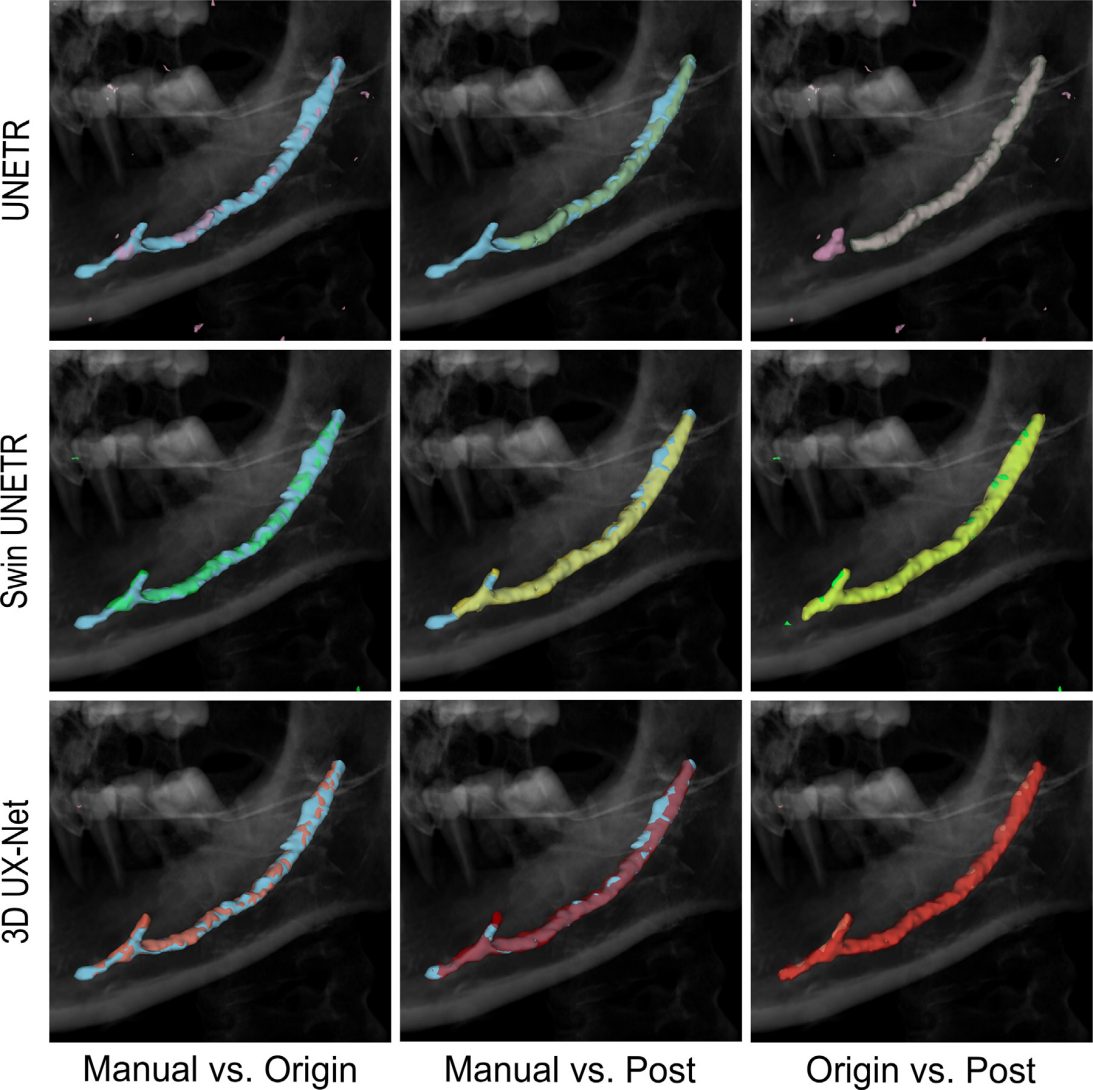
Canal annotations after CBCT segmentation. Note that the manual segmentation results were shown in blue.

All models successfully segmented the anatomical courses of the mandibular, mandibular incisive, and mental canals (Figure 2). Using manual segmentation as a baseline, 3D UX-Net demonstrated superior performance in preserving morphological continuity and spatial coherence. While UNETR often produced fragmented segmentations or erroneously included adjacent structures, 3D UX-Net's output aligned more closely with the underlying anatomy.

Postprocessing consistently improved results across all models. It enhanced the delineation of fine structures, such as canal bifurcations and apical regions, and reduced topological errors like discontinuities and false branches. Visually, postprocessed segmentations appeared more complete and contiguous, with notable improvements in challenging areas like the mandibular incisive canals. This step also reduced the number of misidentified, nonmandibular canal structures. When comparing the postprocessed results, 3D UX-Net maintained its superiority, exhibiting a high degree of completeness and accuracy with fewer misidentifications and a close visual correspondence to the expected canal pathway compared to the other postprocessed models.

### Model performances overall

**Fig. 3:**
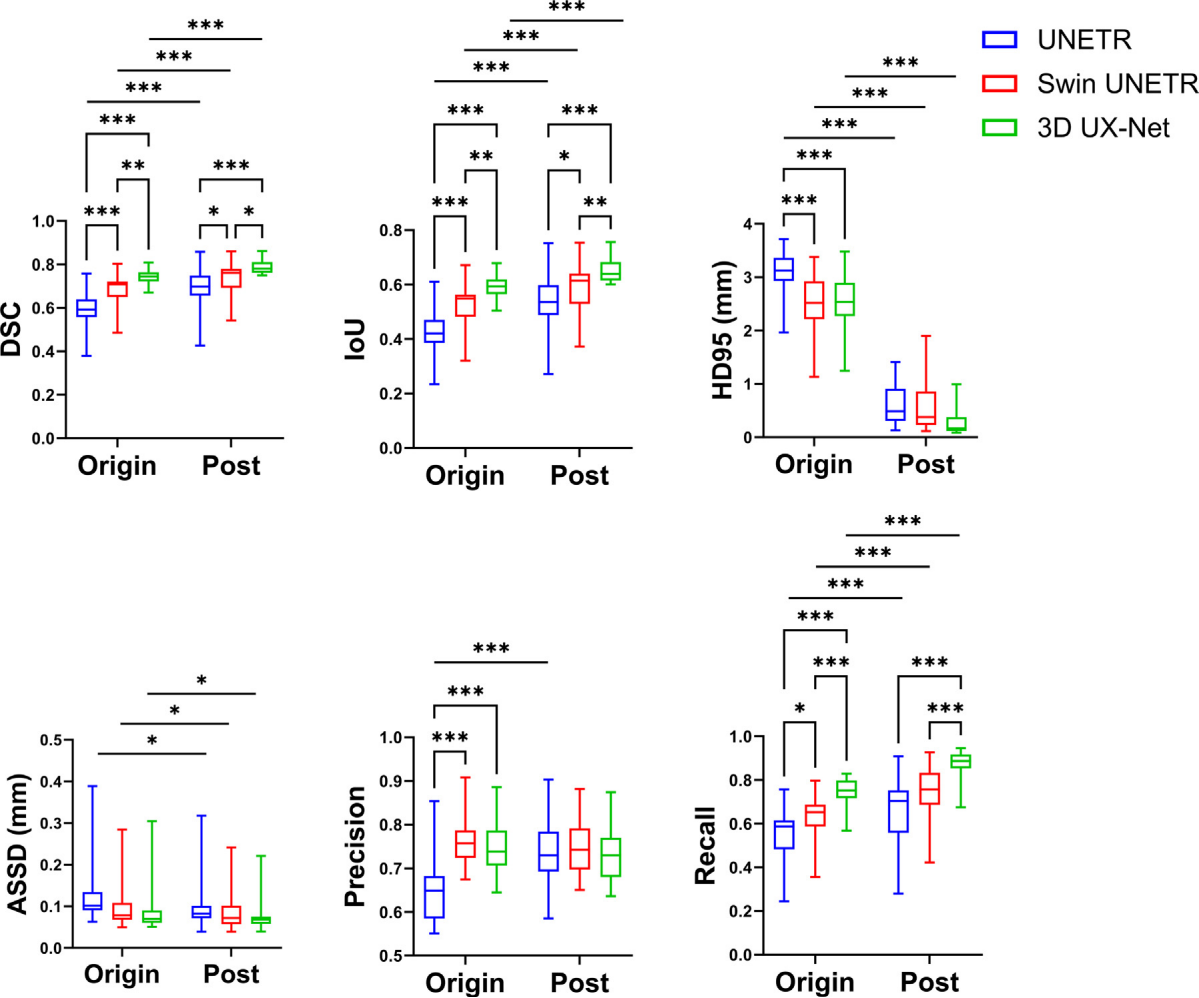
Overall performances.

Overall segmentation performance is shown in Figure 3 and detailed in Supplemental Table S1. In their original forms, both Swin UNETR and 3D UX-Net outperformed UNETR, demonstrating better scores across most metrics, ie, DSC, IoU, HD95, precision, and recall. Specifically, the 3D UX-Net network achieved a higher DSC (*P* < .05), IoU (*P* < .01), and recall (*P* < .001) than Swin UNETR. Furthermore, postprocessing significantly improved the DSC, IoU, HD95, ASSD, and recall for all models. However, it only enhanced the precision of the UNETR network. The 3D UX-Net with postprocessing module achieved the highest segmentation accuracy, yielding a DSC of 0.788, an IoU of 0.652, and HD95 of 0.23 mm, ASSD of 0.083 mm, precision of 72.7%, and recall of 87.0%.

### Model performances on the mandibular and mental canals

**Fig. 4:**
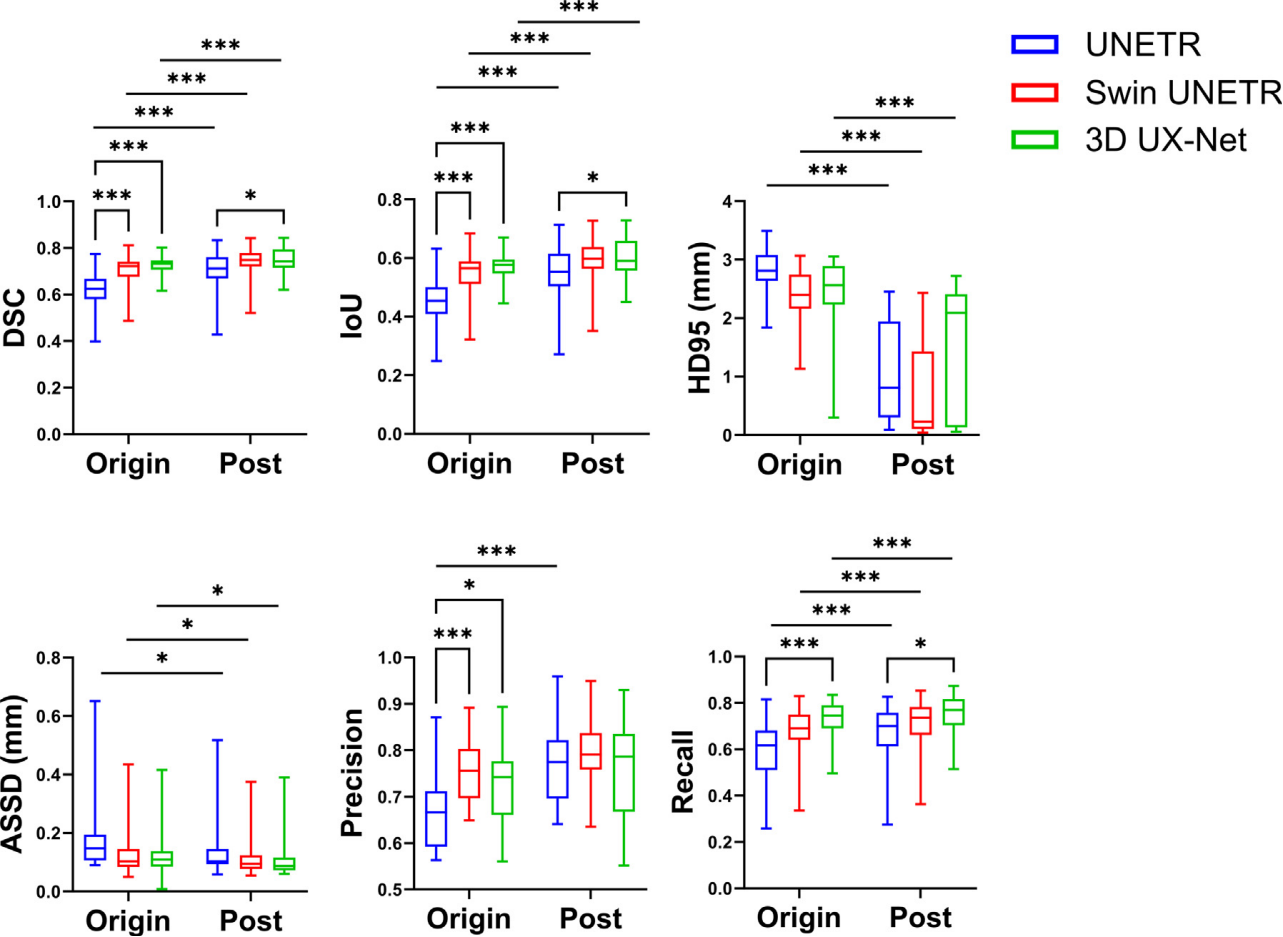
Segmentation performances on the mandibular and mental canals.

Regarding the segmentation of the mandibular and mental canals (Figure 4 and Supplemental Table S2), the original Swin UNETR model showed better DSC, IoU, and precision than UNETR. Additionally, 3D UX-Net demonstrated improved recall compared to UNETR. Postprocessing significantly improved the DSC, IoU, HD95, ASSD, and recall for all models.

### Model performances on the mandibular incisive canals

**Fig. 5:**
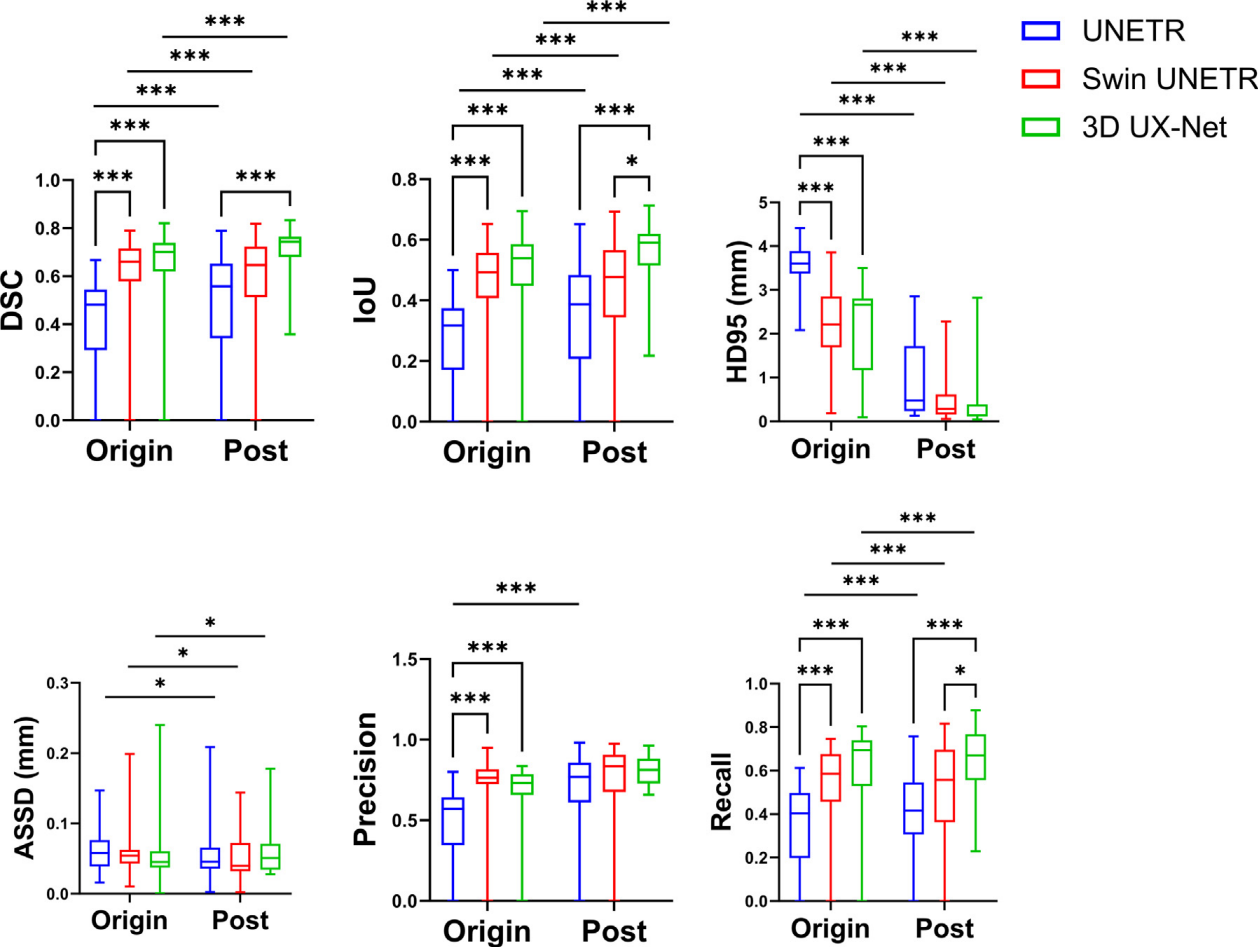
Segmentation performances on the mandibular incisive canals.

For the specific mandibular incisive canals (Figure 5 and Supplemental Table S3), both Swin UNETR and 3D UX-Net networks displayed better DSC, IoU, HD95, precision, and recall than UNETR. Furthermore, the postprocessing procedure significantly improved the DSC, IoU, HD95, ASSD, and recall of all the tested models while only enhancing the precision of the UNETR network.

### Precision-recall curves

**Fig. 6:**
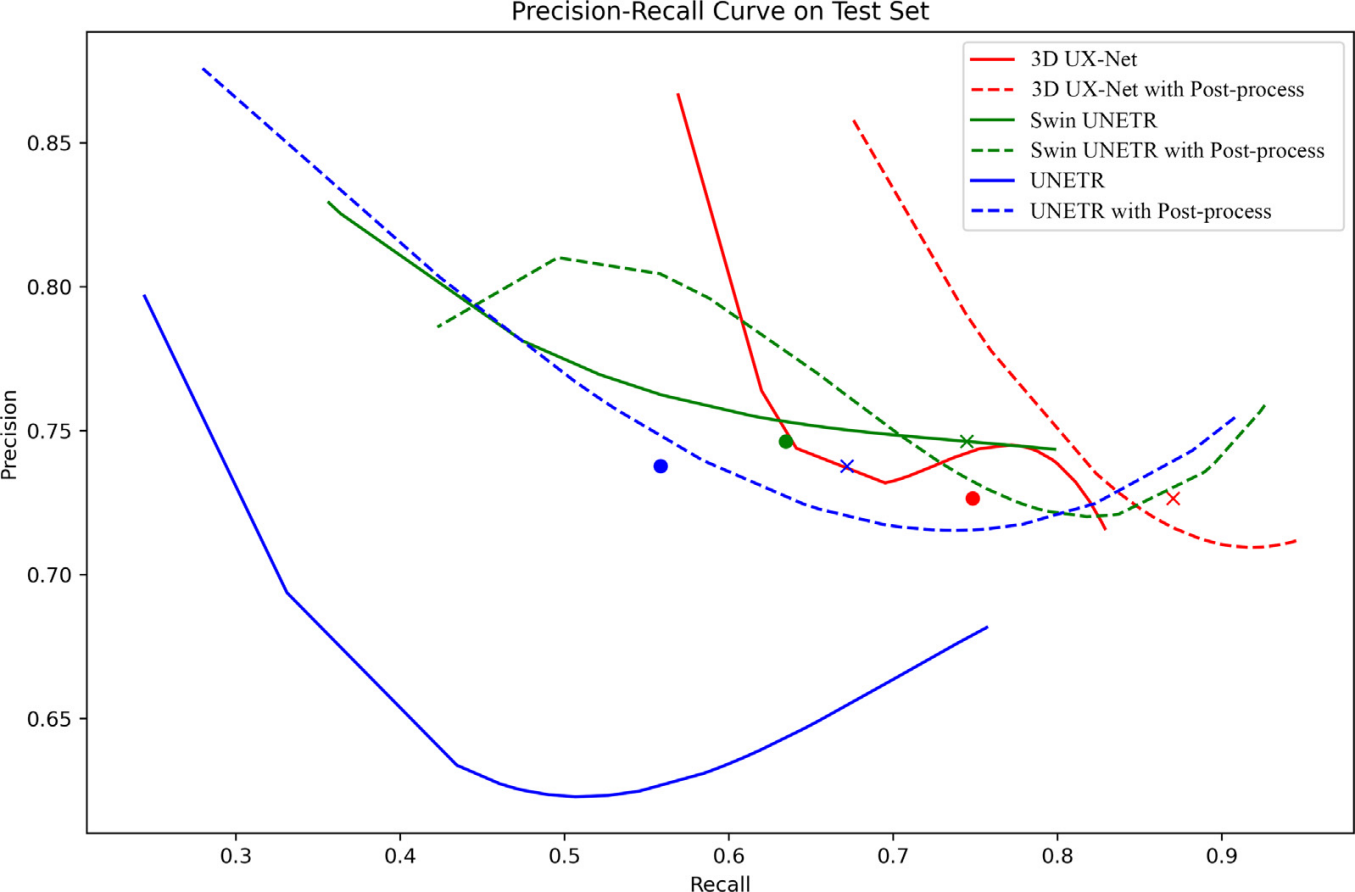
Precision-recall (PR) curves.

An analysis of the precision-recall (PR) curves in Figure 6 offers a deeper look into the performance of the segmentation models. Initially, the baseline configurations of both 3D UX-Net and Swin UNETR were positioned closer to the optimal upper-right corner than UNETR. The application of postprocessing significantly improved the performance of all networks. After this step, both 3D UX-Net and Swin UNETR maintained their advantage over UNETR. Furthermore, 3D UX-Net demonstrated slightly better overall performance than Swin UNETR.

## Discussion

This study evaluated three U-Net-based deep learning models (UNETR, Swin UNETR, and 3D UX-Net) for the segmentation of the mandibular canal and its branches from 173 CBCT scans. All models effectively segmented the mandibular, mandibular incisive, and mental canals, demonstrating excellent boundary agreement and high efficiency, performing at least 25 times faster than manual annotation. Both 3D UX-Net and Swin UNETR consistently outperformed the original UNETR network across most key metrics, including DSC, IoU, HD95, precision, and recall. Notably, 3D UX-Net demonstrated a slight but consistent performance advantage over Swin UNETR in terms of DSC, IoU, and recall. Furthermore, postprocessing based on anatomical characteristics significantly improved the metrics for all models. The combination of 3D UX-Net with the postprocessing module ultimately achieved the highest overall segmentation accuracy, with mean values of 0.788 for DSC, 0.652 for IoU, 0.23 mm for HD95, 0.083 mm for ASSD, 72.7% for precision, and 87.0% for recall.

Several methodological aspects were implemented to ensure a fair and robust evaluation. First, we applied consistent image pre-processing to all datasets before training and evaluation, which minimised variability across the different model architectures.^29^ Second, we incorporated HD95 and ASSD as additional key evaluation metrics, as they are particularly effective for assessing the fine, intricate structures of the mandibular canals.^30^ Finally, we developed a novel postprocessing module that leverages the anatomical characteristics of the canals, which significantly improved the models’ performance.

All models successfully segmented the canals at least 25 times faster than manual annotation. UNETR was the fastest model, followed by Swin UNETR and 3D UX-Net. This performance hierarchy is likely due to each model's computational load, which is measured in floating-point operations (FLOPs) and is the primary determinant of inference time.^31^ UNETR and Swin UNETR both use a transformer architecture, but Swin UNETR’s additional windowing and shifting mechanisms introduce computational overhead, resulting in more FLOPs and a longer runtime than UNETR. In contrast, 3D UX-Net's use of large 3D convolutional kernels for feature extraction results in the highest FLOPs count, leading to the greatest computational demand and the longest runtime of all the models.^32^

Both 3D UX-Net and Swin UNETR surpassed the original UNETR network in performance, with 3D UX-Net demonstrating the most potential. This result may be linked to their architectural designs. The standard vision transformer encoder in UNETR struggles with its global self-attention mechanism, which fails to capture fine-grained local spatial information and ignores the inherent adjacency of image patches—a critical flaw when segmenting intricate structures like the mandibular canal.^14^^,^^33^ In contrast, both Swin UNETR and 3D UX-Net overcome this limitation with a hierarchical design that prioritises local feature extraction.^15^^,^^17^ Swin UNETR uses a windowed multihead self-attention and shifted windows to build a receptive field that expands from local to global, while 3D UX-Net achieves a similar effect by using large-kernel depthwise separable convolutions instead of windowed attention. The slight performance edge of 3D UX-Net over Swin UNETR is likely due to two key architectural choices: its use of depthwise separable convolutions retains the valuable inductive biases of CNN, and its replacement of the standard multilayer perceptron block with depthwise convolutional scaling reduces redundancy in the learned cross-channel context, contributing to its marginal performance gain.

The Swin UNETR and 3D UX-Net demonstrated superior performance over the UNETR network, with their predictions exhibiting a better overlap with manual segmentations, as evidenced by higher DSC and IoU scores. This performance gap is most pronounced when segmenting the intricate mandibular incisive canals. UNETR’s global self-attention mechanism is prone to significant topological errors, such as fragmentation or the inclusion of adjacent bone, because it struggles to capture the fine local spatial details required for tracing such delicate structures. These severe, outlier errors result in a high HD95 score, a metric particularly sensitive to worst-case errors. In contrast, the hierarchical and local feature extraction methods of Swin UNETR and 3D UX-Net preserve morphological continuity, largely avoiding these large-scale errors and consequently yielding a lower HD95. Interestingly, the ASSD metric, which measures the average surface distance, showed no significant difference among the three models. This is because the impact of UNETR’s few substantial errors is diluted when averaged across the entire boundary, making its ASSD score comparable to the other models, whose error profiles consist of many small, consistent deviations. These findings highlight that HD95 and ASSD are particularly valuable metrics for evaluating the segmentation of fine, intricate structures like the mandibular incisive canals. Therefore, we recommend their adoption in future evaluations of similar anatomical features.

Recognising the inherent challenges of mandibular canal segmentation and the propensity of deep learning models to produce minor imperfections (eg, small discontinuities or spurious islands), our study incorporated a tailored postprocessing module. This module combined morphological opening and closing with density-based filtering and proved highly effective: it substantially improved segmentation continuity, eliminated noise, and elevated overall accuracy to the reported levels.^21^^,^^34^ Such postprocessing is widely recognised as a crucial step in medical image segmentation pipelines, refining raw model outputs to achieve clinical usability.^22^ Unlike generic postprocessing, our approach was specifically designed for mandibular canal segmentation. The morphological operations were targeted to enhance connectivity along the canal, while the density-based filtering step was crucial in mitigating the over-segmentation—particularly the expansion sometimes introduced by morphological closing—ensuring closer adherence to the true anatomical boundaries evident in the CBCT data. This novel combination of morphological refinement and density-based correction represents an important contribution, demonstrating its effectiveness in translating high-performing models into clinically reliable tools by addressing specific segmentation failure modes.

Beyond standard overlap metrics, the clinical utility of a segmentation model depends on its boundary precision relative to surgical safety guidelines. In dental implantology, clinicians are universally advised to maintain a safety zone of at least 2 mm between the implant fixture and the neurovascular bundle to avoid iatrogenic nerve injury.^35^ In this context, the HD95 serves as a critical metric for assessing maximum boundary deviation. The 3D UX-Net model yielded an HD95 of 0.23 mm, which is an order of magnitude smaller than the recommended 2 mm safety threshold. Additionally, the ASSD remained consistently low at 0.083 mm. These findings indicate that the model’s automated segmentation boundaries are precise enough to support digital preoperative planning, including virtual implant placement and surgical guide design, without compromising the neurovascular safety zone.

A significant challenge for future studies lies in the accurate segmentation of the incisive nerve canal, especially in regions where the distinct cortical bone boundary becomes indistinct. In such scenarios, AI models must move beyond simple edge detection to the more complex task of differentiating between two nearly isodense regions—the porous trabecular bone and the relatively homogeneous intracanal contents. This ambiguity requires models to possess advanced feature extraction capabilities and a nuanced understanding of spatial relationships, enabling them to infer that a subtle or blurry region is part of a continuous anatomical pathway. The frequent absence of clear local information often leads to segmentation failures, including fragmentation or the misidentification of bone marrow cavities as part of the canal.^36^ To address these challenges, future research should focus on two key areas: developing advanced model architectures, such as Swin UNETR and 3D UX-Net, specifically designed for handling low-contrast boundaries, and creating more sophisticated, anatomy-aware postprocessing algorithms that integrate prior knowledge to correct errors and ensure segmentation continuity for improved clinical usability.

Despite these encouraging results, this study is subject to certain limitations. First, as a single-centre investigation, the generalizability of the findings across different institutions and imaging protocols remains to be confirmed. To mitigate this, we are ensuring the public dissemination of our dataset, which is characterised by meticulous annotations of fine terminal branches. We anticipate that this resource will enable the broader research community to conduct external validation and benchmark future architectures. Second, this study prioritised high-quality data to establish a definitive performance baseline, which necessitated the exclusion of scans with severe metallic artefacts or ambiguous pathologies. Consequently, the models' robustness to real-world clinical heterogeneity—such as beam-hardening artefacts from dental implants or reduced contrast in osteoporotic patients—remains to be fully quantified. Finally, we acknowledge that the current absence of failure case analysis and uncertainty estimation restricts immediate clinical interpretability. Future work will specifically target these gaps by stress-testing the architectures against ‘noisy’ datasets and integrating uncertainty estimation mechanisms to bridge the gap between algorithmic benchmarking and robust clinical deployment.

## Conclusion

Both 3D UX-Net and Swin UNETR significantly outperformed the original UNETR network in segmenting small dental structures. Between the two advanced models, 3D UX-Net demonstrated statistically significant improvements over Swin UNETR in terms of overlap metrics (DSC, IoU) and recall, while maintaining comparable performance in precision and boundary error metrics (HD95, ASSD). The performance of these models can be improved by applying postprocessing procedures based on anatomical characteristics.

## Author contributions

*Investigation, data acquisition, data annotation, formal analysis, data curation, validation, and writing—original draft. H.M.: Methodology, model training, and writing—review and editing*: Ma; *Data acquisition, data annotation, writing—review and editing*: Luo; *Writing—review and editing*: Wang; *Conceptualisation, funding acquisition, resources, and writing—review and editing*: Shao.; *Conceptualisation, supervision, resources, funding acquisition, and review and editing*: Ge; *Conceptualisation, supervision, resources, and review and editing:* Wang.

*Discussed the results, commented on the manuscript, and approved the final version of the manuscript:* All authors*.*

## Ethics statement

This study was approved by the Institutional Review Board (IRB) of the School and Hospital of Stomatology, Shandong University (Approval no. 20241017) and was in accordance with the Declaration of Helsinki for research involving human subjects. The IRB of the School and Hospital of Stomatology, Shandong University waived the need for individual informed consent, and thus, written/verbal informed consent was not obtained from any participant, as this study had a noninterventional retrospective design and all the data were analysed anonymously.

## Funding

This research was supported by the Shandong Province Natural Science Foundation (ZR2024QH137), the Taishan Scholars Program of Shandong Province (tsqn202211325 and tstp20250510), and the National Natural Science Foundation of China (82320108004, 82401120). The funders had no role in the study design, data collection and analysis, decision to publish or preparation of the manuscript. The authors declare that no financial or other potential competing interests exist in this study.

## Data availability

To address the limitations of dataset accessibility and promote reproducibility in dental AI research, we have made the anonymised dataset publicly available. The full dataset, including the raw CBCT scans (in NIfTI format) and the corresponding voxel-level manual annotations, has been deposited in the Hugging Face repository and can be accessed via the following persistent identifier: https://huggingface.co/datasets/mhy0716/mandibular/tree/main.

## Conflict of interest

None disclosed.

## Appendix Supplementary materials

Image, application 1

## Footnotes

Supplementary material associated with this article can be found in the online version at doi:10.1016/j.identj.2026.109427.
