# Supplementary material for: Comparison of Artificial Intelligence Models for Automatic Segmentation of the Mandibular Canals and Branches
Source: Int Dent J. 2026 Feb 6;76(2):109427. doi: 10.1016/j.identj.2026.109427 (PMC12907091; doi:10.1016/j.identj.2026.109427)
Supplement: Supplementary file 1 [file mmc1.docx]

**Supplementary Material**

Supplemental Table S1. Model performances overall

| **Models** | **Parameters** | **Original** | **Post-processed** | **Comparison**  **Origin vs. Post-processed** | **Comparison between groups** | **Original data** | **Post-processed** |
| --- | --- | --- | --- | --- | --- | --- | --- |
| UNETR | DSC | 0.5894 ± 0.0793 | 0.6904 ± 0.0877 | <0.001 | UNETR vs. Swin UNETR | <0.001 | 0.036 |
| Swin UNETR |  | 0.6831 ± 0.0721 | 0.7371 ± 0.0677 | <0.001 | UNETR vs. 3D UX-Net | <0.001 | <0.001 |
| 3D UX-Net |  | 0.7422 ± 0.0356 | 0.7884 ± 0.0323 | <0.001 | Swin UNETR vs. 3D UX-Net | 0.005 | 0.018 |
| UNETR | IOU | 0.4221 ± 0.0780 | 0.5334 ± 0.0979 | <0.001 | UNETR vs. Swin UNETR | <0.001 | 0.027 |
| Swin UNETR |  | 0.5228 ± 0.0792 | 0.5878 ± 0.0821 | <0.001 | UNETR vs. 3D UX-Net | <0.001 | <0.001 |
| 3D UX-Net |  | 0.5914 ± 0.0450 | 0.6519 ± 0.0449 | <0.001 | Swin UNETR vs. 3D UX-Net | 0.004 | 0.007 |
| UNETR | HD95(mm) | 3.0590 ± 0.4177 | 0.5877 ± 0.3677 | <0.001 | UNETR vs. Swin UNETR | <0.001 | 0.995 |
| Swin UNETR |  | 2.5040 ± 0.5003 | 0.5766 ± 0.4757 | <0.001 | UNETR vs. 3D UX-Net | <0.001 | 0.045 |
| 3D UX-Net |  | 2.5590 ± 0.5210 | 0.2924 ± 0.2614 | <0.001 | Swin UNETR vs. 3D UX-Net | 0.894 | 0.056 |
| UNETR | ASSD(mm) | 0.1369 ± 0.0912 | 0.1045 ± 0.0701 | <0.001 | UNETR vs. Swin UNETR | 0.162 | 0.751 |
| Swin UNETR |  | 0.1021 ± 0.0624 | 0.0909 ± 0.0530 | 0.023 | UNETR vs. 3D UX-Net | 0.065 | 0.486 |
| 3D UX-Net |  | 0.0940 ± 0.0660 | 0.0827 ± 0.0518 | 0.022 | Swin UNETR vs. 3D UX-Net | 0.906 | 0.904 |
| UNETR | Precision | 0.6462 ± 0.0705 | 0.7379 ± 0.0702 | <0.001 | UNETR vs. Swin UNETR | <0.001 | 0.875 |
| Swin UNETR |  | 0.7591 ± 0.0550 | 0.7464 ± 0.0579 | 0.436 | UNETR vs. 3D UX-Net | <0.001 | 0.792 |
| 3D UX-Net |  | 0.7427 ± 0.0522 | 0.7266 ± 0.0586 | 0.241 | Swin UNETR vs. 3D UX-Net | 0.609 | 0.489 |
| UNETR | Recall | 0.5583 ± 0.1162 | 0.6719 ± 0.1434 | <0.001 | UNETR vs. Swin UNETR | 0.039 | 0.052 |
| Swin UNETR |  | 0.6345 ± 0.1131 | 0.7446 ± 0.1267 | <0.001 | UNETR vs. 3D UX-Net | <0.001 | <0.001 |
| 3D UX-Net |  | 0.7482 ± 0.0667 | 0.8703 ± 0.0648 | <0.001 | Swin UNETR vs. 3D UX-Net | <0.001 | <0.001 |

Supplemental Table S2. Model performances on the mandibular and mental canals

| **Models** | **Parameters** | **Original** | **Post-processed** | **Comparison**  **Origin vs. Post-processed** | **Comparison between groups** | **Original data** | **Post-processed** |
| --- | --- | --- | --- | --- | --- | --- | --- |
| UNETR | DSC | 0.6185 ± 0.0796 | 0.6982 ± 0.0868 | <0.001 | UNETR vs. Swin UNETR | <0.001 | 0.126 |
| Swin UNETR |  | 0.6944 ± 0.0823 | 0.7378 ± 0.0703 | <0.001 | UNETR vs. 3D UX-Net | <0.001 | 0.035 |
| 3D UX-Net |  | 0.7232 ± 0.0426 | 0.7490 ± 0.0573 | 0.003 | Swin UNETR vs. 3D UX-Net | 0.331 | 0.844 |
| UNETR | IOU | 0.4521 ± 0.0807 | 0.5425 ± 0.0963 | <0.001 | UNETR vs. Swin UNETR | <0.001 | 0.106 |
| Swin UNETR |  | 0.5374 ± 0.0908 | 0.5890 ± 0.0833 | <0.001 | UNETR vs. 3D UX-Net | <0.001 | 0.027 |
| 3D UX-Net |  | 0.5681 ± 0.0512 | 0.6019 ± 0.0724 | <0.001 | Swin UNETR vs. 3D UX-Net | 0.37 | 0.837 |
| UNETR | HD95(mm) | 2.789 ± 0.4379 | 1.092 ± 0.8429 | <0.001 | UNETR vs. Swin UNETR | 0.073 | 0.346 |
| Swin UNETR |  | 2.318 ± 0.5319 | 0.7947 ± 0.8155 | <0.001 | UNETR vs. 3D UX-Net | 0.208 | 0.159 |
| 3D UX-Net |  | 2.427 ± 0.6432 | 1.484 ± 1.064 | <0.001 | Swin UNETR vs. 3D UX-Net | 0.866 | 0.004 |
| UNETR | ASSD(mm) | 0.1953 ± 0.1513 | 0.1469 ± 0.1131 | <0.001 | UNETR vs. Swin UNETR | 0.156 | 0.769 |
| Swin UNETR |  | 0.1391 ± 0.1014 | 0.1260 ± 0.0880 | 0.124 | UNETR vs. 3D UX-Net | 0.11 | 0.588 |
| 3D UX-Net |  | 0.1338 ± 0.0915 | 0.1171 ± 0.0831 | 0.031 | Swin UNETR vs. 3D UX-Net | 0.984 | 0.954 |
| UNETR | Precision | 0.6642 ± 0.0727 | 0.7755 ± 0.0898 | <0.001 | UNETR vs. Swin UNETR | <0.001 | 0.559 |
| Swin UNETR |  | 0.7527 ± 0.0631 | 0.7994 ± 0.0799 | <0.001 | UNETR vs. 3D UX-Net | 0.033 | 0.822 |
| 3D UX-Net |  | 0.7231 ± 0.0788 | 0.7616 ± 0.1024 | <0.001 | Swin UNETR vs. 3D UX-Net | 0.41 | 0.238 |
| UNETR | Recall | 0.601 ± 0.131 | 0.6616 ± 0.1384 | <0.001 | UNETR vs. Swin UNETR | 0.144 | 0.405 |
| Swin UNETR |  | 0.6631 ± 0.1307 | 0.7038 ± 0.1196 | 0.013 | UNETR vs. 3D UX-Net | <0.001 | 0.015 |
| 3D UX-Net |  | 0.735 ± 0.0716 | 0.754 ± 0.0897 | 0.434 | Swin UNETR vs. 3D UX-Net | 0.077 | 0.281 |

Supplemental Table S3. Model performances on the mandibular incisive canals

| **Models** | **Parameters** | **Original** | **Post-processed** | **Comparison**  **Origin vs. Post-processed** | **Comparison between groups** | **Original data** | **Post-processed** |
| --- | --- | --- | --- | --- | --- | --- | --- |
| UNETR | DSC | 0.3867±0.1996 | 0.4989±0.2123 | 0.002 | UNETR vs. Swin UNETR | <0.001 | 0.155 |
| Swin UNETR |  | 0.6191±0.1661 | 0.5921±0.1967 | 0.785 | UNETR vs. 3D UX-Net | <0.001 | <0.001 |
| 3D UX-Net |  | 0.6492±0.1609 | 0.7046±0.1072 | 0.239 | Swin UNETR vs. 3D UX-Net | 0.821 | 0.068 |
| UNETR | IOU | 0.2568±0.1445 | 0.3558±0.1746 | 0.001 | UNETR vs. Swin UNETR | <0.001 | 0.1 |
| Swin UNETR |  | 0.4648±0.1440 | 0.4437±0.1743 | 0.82 | UNETR vs. 3D UX-Net | <0.001 | <0.001 |
| 3D UX-Net |  | 0.4964±0.1407 | 0.553±0.1144 | 0.113 | Swin UNETR vs. 3D UX-Net | 0.737 | 0.03 |
| UNETR | HD95(mm) | 3.561±0.5076 | 0.9995±0.9460 | <0.001 | UNETR vs. Swin UNETR | <0.001 | 0.11 |
| Swin UNETR |  | 2.227±0.9319 | 0.5347±0.6174 | <0.001 | UNETR vs. 3D UX-Net | <0.001 | 0.01 |
| 3D UX-Net |  | 2.161±1.014 | 0.3252±0.5446 | <0.001 | Swin UNETR vs. 3D UX-Net | 0.954 | 0.62 |
| UNETR | ASSD(mm) | 0.0630±0.0314 | 0.0616±0.0475 | 0.992 | UNETR vs. Swin UNETR | 0.998 | 0.643 |
| Swin UNETR |  | 0.0637±0.0387 | 0.0516±0.0329 | 0.1 | UNETR vs. 3D UX-Net | 0.947 | 0.984 |
| 3D UX-Net |  | 0.0595±0.0465 | 0.0596±0.0352 | >0.999 | Swin UNETR vs. 3D UX-Net | 0.925 | 0.75 |
| UNETR | Precision | 0.4860±0.2465 | 0.6867±0.2712 | <0.001 | UNETR vs. Swin UNETR | <0.001 | 0.875 |
| Swin UNETR |  | 0.7465±0.1663 | 0.7813±0.2022 | 0.436 | UNETR vs. 3D UX-Net | <0.001 | 0.792 |
| 3D UX-Net |  | 0.6974±0.1639 | 0.8101±0.0862 | 0.241 | Swin UNETR vs. 3D UX-Net | 0.609 | 0.489 |
| UNETR | Recall | 0.3425±0.1814 | 0.4177±0.1990 | 0.051 | UNETR vs. Swin UNETR | <0.001 | 0.146 |
| Swin UNETR |  | 0.5485±0.1823 | 0.5176±0.2160 | 0.684 | UNETR vs. 3D UX-Net | <0.001 | <0.001 |
| 3D UX-Net |  | 0.6195±0.1782 | 0.6512±0.1603 | 0.667 | Swin UNETR vs. 3D UX-Net | 0.374 | 0.034 |
